# Bickerstaff’s brainstem encephalitis: a rare case of neurologic complication in Ulcerative Colitis

**DOI:** 10.1186/s12883-023-03430-0

**Published:** 2023-10-26

**Authors:** Haram Joo, Chung Seok Lee, Sangwon Joe, Jinu Han, Han-Kyeol Kim, Hanna Cho

**Affiliations:** 1 Department of Neurology, Yonsei University College of Medicine, Gangnam Severance Hospital, Seoul, South Korea; 2 Department of Ophthalmology, Yonsei University College of Medicine, Seoul, South Korea

**Keywords:** Autoimmune encephalitis, Encephalitis, Bickerstaff’s brainstem encephalitis, Ulcerative Colitis, Mesalazine, Inflammatory bowel disorder

## Abstract

Bickerstaff’s brainstem encephalitis is a rare autoimmune disorder that presents with ataxia, ophthalmoplegia, disturbance of consciousness and quadriplegia. A 45-year-old man with a history of ulcerative colitis (UC) taking mesalazine (5-aminosalicylic acid) visited the emergency room presenting with ataxia, ophthalmoplegia and a progressively worsening cognitive impairment. Cerebrospinal fluid analysis showed mild elevation in protein and white blood cell count and increased intracranial pressure. Anti-GQ1b autoantibodies were found positive in the patient’s serum and contrast-enhanced brain magnetic resonance imaging showed diffuse leptomeningeal enhancement and pontine lesions. Based on these findings and the patient’s clinical course and history, he was diagnosed with Bickerstaff’s brainstem encephalitis. Mesalazine was discontinued and high-dose steroid pulse therapy was started, followed by intravenous immunoglobulin, which resulted in gradual improvement of the neurologic symptoms. When an ulcerative colitis patient presents with progressive cognitive impairment, quadriplegia and disturbance of consciousness and gait, Bickerstaff brainstem encephalitis should be considered in the differential diagnosis and prompt immunotherapy may lead to favorable prognosis.

## Introduction

Ulcerative colitis (UC) is an inflammatory disorder of the digestive tract characterized by severe inflammation mainly involving the colon and rectum. Among the extraintestinal manifestations of UC, neurologic complications are extremely rare – one retrospective study estimated the incidence of neurological complications of inflammatory bowel disorders (IBD) to be 3% [1]. Among UC patients, the most common neurologic complications are peripheral neuropathy, cerebrovascular disease and demyelinating disease [2]. With the increasing use of biologic agents such as infliximab and immunosuppressants, there seems to be an increasing incidence of IBD-associated neuroinflammatory disorders including progressive multifocal leukoencephalopathy and autoimmune encephalitis [3–6].

Bickerstaff’s brainstem encephalitis (BBE) is an immunologic condition involving inflammation of the central nervous system that presents with ataxia, external ophthalmoplegia, disturbance of consciousness and quadriplegia with a positive serum GQ1b antibody and relevant MRI findings suggestive of autoimmune encephalitis [7, 8]. In contrast to the more commonly encountered neurologic complications, BBE is rarely reported in UC patients. Here, we present this rare neurologic complication in a UC patient.

## Case presentation

A 45-year-old male with UC presented to the emergency room for drowsy mentality, blurred vision, dysarthria, and gait disturbance. He was diagnosed with UC several years ago and was taking mesalazine as maintenance treatment. Three months prior to admission, he reported of losing significant amounts of weight, worsening visual acuity and a new-onset headache. One month before admission, he became dysarthric with progressive cognitive impairment. He gradually became confused and inattentive. The day before admission, he began to have trouble swallowing food. In the emergency room, on neurologic exam, the patient appeared slightly drowsy, unable to articulate properly and had difficulty swallowing food with reduced gag reflex. He complained of blurry vision without any visual field defects. Further examination of extraocular muscles revealed bilateral abducens nerve palsy and cerebellar exam showed ataxic limbs.

**Fig. 1:**
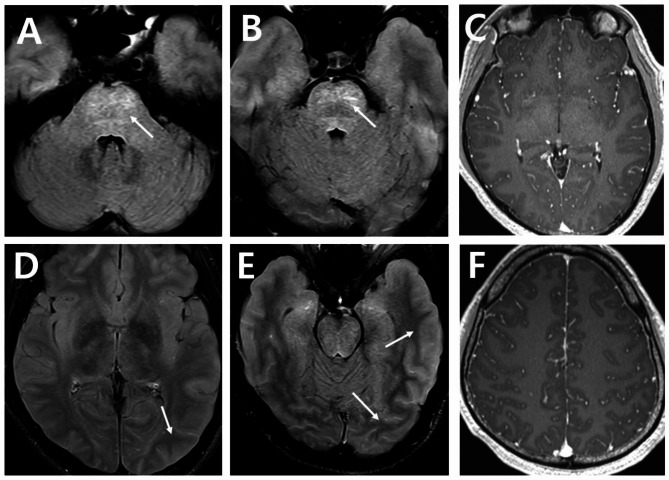
Initial brain magnetic resonance imaging with gadolinium-enhancement. (**A**) (**B**) FLAIR imaging with gadolinium-enhancement revealed subtle high signal intensity in the central pons suggestive of meningoencephalitis (**D**) (**E**) FLAIR imaging with gadolinium-enhancement revealed diffuse leptomeningeal enhancement combined with diffuse cortical swelling and crowding gyri suggestive of increased intracranial pressure and meningoencephalitis (**C**) (**F**) T1-weighted gadolinium-enhancement revealed diffuse leptomeningeal enhancement

**Fig. 2:**
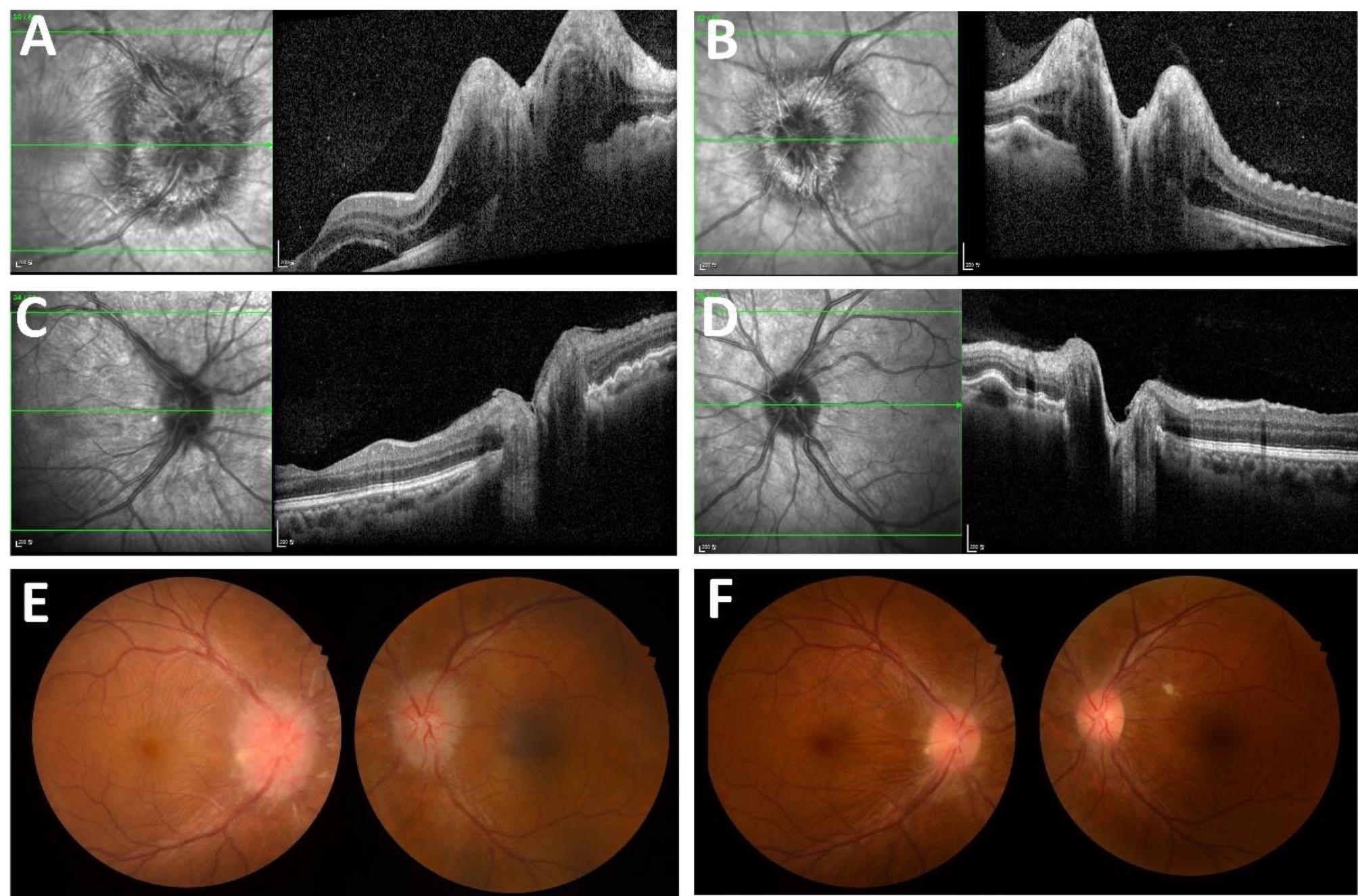
Initial and post-treatment ophthalmologic evaluation including fundoscopic imaging and optical coherence tomography. (**A**) (**B**) On admission, initial optical coherence tomography (OCT) imaging revealed severe disc swelling along with excessive subretinal fluid collection (**C**) (**D**) Post-immunotherapy follow-up OCT imaging revealed improved disc swelling and marked improvement in the subretinal fluid collection (**E**) Initial fundoscopic imaging revealed severe disc swelling suggestive of papilledema (**F**) Post-treatment fundoscopic exam showed marked improvement of disc swelling and papilledema

Initial brain computed tomography (CT) scan revealed crowding of gyri. Lumbar puncture tests showed cerebral spinal fluid (CSF) opening pressure of 280 mm of water with normal glucose level (59 mg/dL), elevated protein level (74.5 mg/dL) and slightly elevated white blood cell count (13 total nucleated cells with mononuclear dominancy (92.3%)). Brain magnetic resonance image (MRI) showed diffuse cortical swelling and crowding gyri and leptomeningeal enhancement with T2-hyperintense lesions seen on the pons indicative of parenchymal brainstem inflammation (Fig. 1). Fundoscopic examination revealed papilledema and optic disc swelling with subretinal fluid collection seen in optical coherence tomography (Fig. 2). Electromyography and nerve conduction studies were all normal.

With the initial diagnosis of infectious rhombencephalitis, first-line treatment included intravenous acyclovir, ceftriaxone and vancomycin. Considering the possibility of drug-induced encephalopathy, mesalazine was promptly discontinued. Furthermore, to control the increased intracranial pressure, mannitol infusion was started subsequently. After a week of antibiotics and anti-viral therapy, there was no noticeable improvement and additional laboratory tests on viral and fungal antibodies in the CSF and serum along with a multiple bacterial polymerase chain reaction (PCR) panel including *Listeria monocytogenes* did not yield any positive results. On the second week of treatment, a comprehensive work-up of autoimmune encephalitis was performed which was positive for serum GQ1b antibody. Under the impression of BBE, five days of intravenous high dose methylprednisolone (1 g/day) was given, followed by five days of intravenous immunoglobulin (400 mg/kg/day). On the second day of immunoglobulin therapy, he was reporting of improved dysarthria. On the day of discharge, he was alert and able to walk with improved balance. On one-month follow-up, he was completely back to his baseline state. With mesalazine discontinued, he has remained symptom free for two years.

## Discussion

BBE is an immunologic disorder that presents with external ophthalmoplegia, disturbance of consciousness, quadriplegia and ataxia with hyper-reflexia. The sensitivity of serum anti-GQ1b antibodies is between 60 and 70% for Bickerstaff encephalitis, with very high specificity of over 90%.^8^ The mainstay treatments for BBE are high dose intravenous methylprednisolone, intravenous immunoglobulin and plasmapheresis [9]. Our patient was started on high-dose steroid therapy followed by intravenous immunoglobulin, resulting in improvement of the neurologic symptoms.

Our case illustrates an IBD with a proposed immunologic etiology presenting as a rare neurologic manifestation, autoimmune encephalitis. Most neurologic complications of UC have been reports on peripheral neuropathies or central nervous system (CNS) demyelinating diseases. In one observational study including 772 patients with IBD, the cumulative incidence of peripheral neuropathy was 0.7% after 10 years [10]. As far as is known, there are only rare reports of autoimmune encephalitis in association with UC in the literature. A previous report presented a 25-year-old UC patient who initially had digestive problems later to develop into generalized dysesthesia and deterioration of consciousness which was eventually diagnosed as anti-GQ1b antibody positive BBE [7]. The patient received plasmapheresis, which also resulted in gradual improvement of neurologic symptoms.

One pathophysiological mechanism of UC has been suggested to be an immune dysregulation of gut microbiota [11]. Further investigation into the dysfunction of the gut-brain-axis could enhance our understanding of the relationship between autoimmune central nervous system disorders and UC. According to a report on a patient diagnosed with an UC-associated CNS disorder, an autoimmune vasculitic etiology was proposed on examination of the patient’s skin biopsy [12].

A recent report on an autoimmune encephalitis caused by prolonged use of a biologic agent, infliximab, suggests a drug-induced autoimmune dysregulation mechanism in the CNS [5]. Mesalazine, a non-biologic agent commonly prescribed for its immunomodulatory actions, has previously been associated with adverse neurologic events such as transverse myelitis or encephalopathy in patients with UC [13–15]. Similar to all non-biological agents, there is certainly a possibility that a new neurological event, autoimmune encephalitis, may be attributed to the prescribed medicine. In line with previous reports of intracranial hypertension in UC patients taking mesalazine, the papilledema seen in our patient may also be a result of a drug-induced adverse event. Since the day of admission, mesalazine has been discontinued from the treatment regimen and our patient has remained clinically stable at the one-year follow-up with no relapse.

While IBD is an inflammatory disorder of the digestive system, an acute neurologic symptom may warrant the need for a proper diagnostic work-up in collaboration with a multidisciplinary team to recognize a potentially life-threatening extra-intestinal manifestation of UC, autoimmune encephalitis. When an UC patient presents with cognitive dysfunction, external ophthalmoplegia and disturbance of consciousness and gait, BBE should be considered in the differential diagnosis and prompt initiation of immunotherapy with discontinuation of mesalazine may lead to a favorable prognosis.

## Data Availability

All data used in this study are available from the corresponding author on reasonable request.
